# The Clinicopathological Impact of Granulocyte-Macrophage Colony-Stimulating Factor Gene Expression and Different Molecular Prognostic Biomarkers in Egyptian Acute Myeloid Leukemia Patients

**DOI:** 10.31557/APJCP.2020.21.7.1993

**Published:** 2020-07

**Authors:** Bassant Nagdy, Hebatallah A Kassem, Abdel-Rahman B Abdel-Ghaffar, Dina M Seoudi, Neemat M Kassem

**Affiliations:** 1 *Molecular Oncology Unit, Kasr Al-Aiby Centre of Clinical Oncology; Nuclear Medicine, School of Medicine, Cairo University, Egypt. *; 2 *Department of Clinical and Chemical Pathology, Kasr Al Ainy Centre of Clinical Oncology, Nuclear Medicine, School of Medicine, Cairo University, Cairo, Egypt.*; 3 *Department of Biochemistry, Faculty of Science, Ain Shams University, Cairo, Egypt. *

**Keywords:** AML, GM-CSF, CEBPA, FLT3-ITD, NPM1 mutation A

## Abstract

**Background:**

Acute myeloid leukemia (AML) is characterized by clonal expansion of myeloid precursors with diminished capacity for differentiation. It develops as the consequence of a series of genetic changes in a hematopoietic precursor cell. Purpose This study aimed to investigate the correlation between *GM-CSF* gene expression and different molecular prognostic markers such as *FLT3-ITD*, *NPM1* mutation A and *CEBPA* gene expression in 100 Egyptian AML patients. As well as, correlation with the response to induction therapy, DFS andOS in these patients.

**Methodology:**

Quantitative assessment of *GM-CSF* gene expression was performed by qRT-PCR. Additional prognostic molecular markers were determined as FLT3-ITD and NPM1 mutation A together with quantitative assessment of *CEBPA* gene expression by qRT-PCR.

**Results:**

Patients with high GM-CSF expression levels had better OS and DFS with p value 0.004 and 0.02, respectively. However, no statistically significant difference between low andhigh *GM-CSF* gene expression was found regarding the response to therapy (p value= 0.08). Most patients with low *CEBPA* expression had resistant disease together with poor OS and DFS (P value = <0.001 for each). Our results showed that patients with high *CEBPA* gene expression whether *GM-CSF* gene expression was high or low had significant higher complete remission rates (p value = 0.1 for each). However, low *GM-CSF* gene expression andlow *CEBPA* gene expression showed poor response to treatment.

**Conclusion:**

Our findings suggest that molecular diagnostic biomarkers for AML are an essential tool that improves prognostication andhence better patients’ management.

## Introduction

Acute myeloid leukemia (AML) is a heterogeneous hematological disorder, characterized by clonal expansion of myeloid precursors with diminished capacity for differentiation resulting in an accumulation of large numbers of abnormal, immature myeloid cells (Hussein et al., 2019). The age-adjusted incidence of AML is 4.3 per 100,000 annually in the United States (US). Incidence increases with age with a median age at diagnosis of 68 years in the US (Shallis et al., 2019). In Egypt, leukemia is the most common presented hematological malignancy (75%), nearly half of leukemic cases were acute myeloid leukemia which develops as the consequence of a series of genetic changes in a hematopoietic precursor cell (Hussein et al., 2019). Many somatic acquired mutations have been identified in AML with normal karyotype such as *FLT3-ITD, NPM1, CEBPA*…etc. The mutation in FMS-like tyrosine kinase 3 (*FLT3*) gene is one of the most common genetic abnormalities found in AML patients. The cytogenetic location of *FLT3* gene is on chromosome 13q12.2 and is a member of the class III receptor tyrosine kinase (RTK). The *FLT3* mutation results in constitutive activation of the receptor with independent dimerization of FLT3 ligand (FL), and auto-phosphorylation which result in uncontrolled proliferation and apoptosis (Kumsaen et al., 2016). Mutations in *FLT*3 gene have been identified in two functional domains of the receptor, internal tandem duplications (ITDs) in the juxtamembrane domain (JM) and activating point mutations in the second tyrosine kinase domain (TKD). *FLT3-ITD* mutation is present in ~ 20-30% of adult AML patients and 5-15% of pediatric AML patients (Faiz and Rashid, 2019). AML patients with the *FLT3- ITD* mutation have increased risk of relapse, decreased DFS and OS rates. Also, it is currently used as a molecular prognostic marker for risk classification strategies (Port et al., 2014). Among the genetic alterations, a potential prognostic genetic marker is the nucleophosmin 1 (*NPM1*) gene which is located on chromosome 5q35.1 and the protein encoded by this gene is involved in several cellular processes, including centrosome duplication, protein chaperoning, and cell proliferation (Sportoletti et al., 2015). Mutations in *NPM1* are detected in 20-30% of AML patients, as well as in 50-60% of AML patients with normal karyotype. The presence of NPM1 mutations in AML is associated with favorable outcomes when treated with intensive chemotherapy, especially in the absence of *DNTM3A *and *FLT3-ITD* mutations. Also, a greater chemosensitivity of NPM1- mutated compared with NPM1–wild-type leukemic blasts was found (Montalban-Bravo et al., 2019). The CCATT enhancer binding protein alpha (*CEBPA*) transcription factor is an important regulator of myeloid cells proliferation and differentiation. *CEBPA* consists of an N-terminal transcriptional activation domain and a C-terminal basic leucine zipper (bZIP) domain. *CEBPA* mutations are found in 5-14% of AML patients especially M1, M2, or in some cases M4 (Abou-Elella et al., 2019). These mutations can be classified into 2 types: one is an N-terminal frame-shift mutation disrupting p42 and producing p30 as a major product, and the other is a C-terminal in-frame mutation disrupting the bZIP region. Most AML patients with CEBPA mutations have both mutations simultaneously, and such patients have a favorable outcome (Wouters et al., 2009). Granulocyte macrophage colony-stimulating factor (*GM-CSF*) plays a critical role in myeloid differentiation and in several immune and inflammatory processes. The human *GM-CSF *gene is ~ 2.5 kbp which is located in close proximity to the *interleukin 3* gene within a T helper type 2-associated cytokine gene cluster at chromosome region 5q31, which is known to be associated with interstitial deletions in the 5q- syndrome and AML cases (Bowers et al., 2009). This study aimed to investigate the correlation between* GM-CSF* gene expression and different molecular prognostic markers such as *FLT3-ITD*, *NPM1* mutation A and *CEBPA* gene expression in Egyptian acute myeloid leukemia patients. As well as, correlation with the response to therapy, disease free survival (DFS) and overall survival (OS) in these patients.

## Materials and Methods

*Subject and methods*

*Study population*

The present study included 100 AML patients and their ages ranged between 12 and 77 years. They were selected in the period from 2016 to 2018. Twenty age and sex matched healthy volunteers were included in the current study as control group. For patients and controls, 2 ml EDTA blood samples was collected under complete aseptic conditions for molecular studies. 

*RNA extraction*

Extraction of total RNA was performed by QIAamp RNA Blood Mini Kit (Qiagen, Germany) according to manufacturer’s instructions. RNA integrity was tested on the Nanodrop (ND-1000) and stored at −80^o^C. Total RNA was reverse transcribed using random primers with a high-capacity cDNA archive kit (Applied Biosystem, Foster city, CA, USA).

*Detection of FLT3/ITDs and NPM1 mutation A*

The *FLT3* gene was detected using Forward primer: 5’CATTGTCGTTTTAACCCTGCTA3’ and Reverse primer: 5’ATATTCTCGTGGCTTCCCAG 3’. The PCR reaction was done as described by Lilakos et al., (2006) with 360-bp fragment visualized on a 3% agarose gel. For type A mutation in *NPM1* exon 12 detection, we used forward primer: 5’CCAAGAGGCTATTCAAGATCTCTCTC3’ and reverse primer: 5’ACCATTTCCATGTCTGAGCACC3’according to Ottone et al., 2008 with 320-bp fragment visualized by electrophoresis on 2% agarose gel. An internal control; *ABL* was used with primers sequence: 5’GCATCTGACTTTGAGCCTCAG3’ and5’TGACTGGCGTGATGTAGTTGCTT3’ with same PCR conditions and 258 bp fragment visualized on 2% agarose gel.

*Quantitative assessment of CEBPA gene expression*

*CEBPA* gene expression was tested by real time PCR using Taqman primer and probe sets on StepOne machine (Applied Biosystems, USA). We used the following primer sequences for *CEBPA *gene: F: 5’-TCGGTGGACAAGAACAG-3’, R: 5’GCAGGCGGTCATT-3’, and the probe ([6-FAM]-ACAAGGCCAAGCAGCGC-[TAMRA-6-FAM]). Commercially available primers and probe for reference *GAPDH* gene were used for normalization and this probe was labeled with VIC dye. The PCR reaction was done as described by Kassem et al., (2013). The relative quantification (RQ) of *CEBPA* gene expression was assessed by 2^−ΔΔCt^ method (ΔΔCt = {[Ct(CEBPA sample) − Ct(GAPDH sample)] − [Ct(CEBPA control) – Ct (GAPDH control)]}. 

*Quantitative assessment of GM-CSF gene expression*

*GM-CSF* gene expression was tested by real time PCR using Taqman technology on StepOne machine (Applied Biosystems, USA). We used the following primer sequences for *GM-CSF* gene: forward primer 5’-CTGCTGAGATGAATGAAACAG-3’and reverse primer 5’-TCCAAGATGACCATCCTGAG-3’; FAM (6-carboxy fluorescein) probe 5’-ACTCCCACCATGGCTGTGG-3’ (TaqMan GMCSF, access no. M11220, Applied Biosystems). Commercially available primers and probes for reference *GAPDH* gene were used for normalization and this probe was labeled with VIC dye. The PCR reaction was done as described by Kassem et al., (2018). The relative quantification (RQ) of *GM-CSF* gene expression was assessed by 2^−ΔΔCt ^method (ΔΔCt = {[Ct(GM-CSF sample) − Ct(GAPDH sample)] − [Ct(GM-CSF control) – Ct (GAPDH control)]}.

*Data analysis*

Data was analyzed using IBM^©^ SPSS^©^ Statistics version 22 (IBM^©^ Corp., Armonk, NY, USA). Numerical data were expressed as mean and standard deviation or median and range as appropriate. Qualitative data were expressed as frequency and percentage. Chi-square test or Fisher’s exact test was used to examine the relation between qualitative variables. For not normally distributed quantitative data, comparison between two groups was done using Mann-Whitney test (non-parametric t-test). Spearman-rho method was used to test correlation between numerical variables. Survival analysis was done using Kaplan-Meier method and comparison between two survival curves was done using log-rank test. All tests were two-tailed. A p-value < 0.05 was considered significant.

## Results

*Patients’ characteristics*

Our patients were 48 males and 52 females and their ages ranged between 12 and 77 years with a mean value of 37.3±14.9 years. As regards FAB classification, 1% was M0, 23% were M1, 17% were M2, 27% were M3, 12% were M4, 15% were M5, 3% were M6 and 2% M7. Molecular studies revealed 37 patients had *FLT3/ITD* mutation and 36 patients had *NPM1* mutation A. The cytogenetic patients studies showed 15 patients were t(8;21) positive, 8 patients were inv.16 positive and 27 patients were t (15;17) positive. 

*CEBPA gene expression levels*

AML patients with *CEBPA* gene expression level below cut off value which was the mean expression level in the control group (1.13) were considered as “CEBPA low expression”, while those with expression level higher than (1.13) were considered as “*CEBPA* high expression”. The majority of patients (59/100) showed low *CEBPA* expression levels ranged between 0.0013 and 0.99, a mean value of 0.28 ± 0.35 and median value of 0.08. In forty-one cases, higher expression levels were recorded with a range of 1.15 and 1.99, a mean value of 1.68 ± 0.21 and median value of 1.68. Statistical analysis showed significant difference in expression levels between the two groups with p value < 0.001. Comparison between AML patients with low versus high *CEBPA* gene expression according to their clinical and laboratory data was described in Table 1. 

*GM-CSF gene expression levels*

Patients with *GM-CSF* gene expression level below cut off value which was the mean expression level in the control group (1.07) were considered as “GM-CSF low expression”, while those with expression level higher than (1.07) were considered as “GM-CSF high expression”. Many of the patients (55/100) showed low GM-CSF expression levels ranged between 0.001 - 0.99, a mean value of 0.27 ± 0.35 and median value of 0.1. In forty-five cases, higher expression levels were recorded with a range of 1.08 and 2.88, a mean value of 1.87 ± 0.41 and median value of 1.88. Statistical analysis showed significant difference in expression levels between the two groups with p value < 0.001. Comparison between AML patients with low versus high GM-CSF expression according to their clinical and laboratory data was described in Table 2. 

*Correlation between CEBPA and GM-CSF gene expression levels, FIT3/ITD and NPM1 mut. A and response to therapy*

Complete remission (CR) was defined as recovery of bone marrow morphology with less than 5% blasts, neutrophil count 1/109/L or more, platelet count 100/109/L or more, and no evidence of extra medullary leukemia. Resistant disease (RD) was defined as treatment resistance when evaluation did not meet the criteria of complete remission. Early death was defined as death before completion of the induction therapy cycle. These latter patients were not included in evaluation of resistant disease. Accordingly, only 55 patients were evaluated for response to induction therapy. Twenty-eight patients achieved *CR* with *CEBPA* gene expression level ranged between 0.88 and 1.99, with a mean value of 1.69 ± 0.24 and median value of 1.69. Twenty-seven patients had *RD* with *CEBPA* gene expression level ranged between 0.003 and 1.98, with a mean value of 0.92 ± 0.73 and median value of 0.88. There was statistically significant difference noticed in *CEBPA* gene expression between the two patients’ groups with P value <0.001. Regarding high *CEBPA* expression patients, 27 patients had achieved CR after induction therapy, while 12 patients had RD. However, patients with low *CEBPA* expression, only one patient achieved CR after induction therapy while, 15 patients had RD. A statistically significant difference was found between high andlow *CEBPA* expression with P value <0.001, where higher number of patients achieved *CR* had high gene expression levels. In 28 patients who achieved *CR*, *GM-CSF* gene expression level ranged between 0.03 and 2.88, with a mean value of 1.32 ± 0.89 and median value of 1.68. In patients with *RD*,* GM-CSF* gene expression level ranged between 0.01 and 2.65, with a mean value of 0.86 ± 0.85 and median value of 0.54. There was statistically significant difference noticed in *GM-CSF* gene expression between the two patients’ groups with P value = 0.04. Regarding high *GM-CSF *expression patients, 17 patients achieved CR after induction therapy, while 10 patients had RD. However, in low *GM-CSF* expression patients, 11 patients achieved CR while, 17 patients had RD. There was no statistically significant difference between patients with high and low *GM-CSF* gene expression as regards the response to therapy with P value = 0.08. Also, we tried to verify the impact of* FLT3/ITD* and *NPM1* mutations on the response to therapy, *FLT3/ITD* showed no statistically significant difference between cases who achieved CR and those with RD with p-value = 0.11 where, CR rates were higher in patients with wild *FLT3/ITD*. As regards *NPM1 *mut. A, no statistically significant difference was found between cases who achieved CR and those with RD with p value = 0.22. Finally, CR rates were higher in patients with high* CEBPA* gene expression whether *GM-CSF* gene expression levels were high or low as shown in Table 3. 

*Correlation between CEBPA and GM-CSF gene expression levels, FIT3/ITD and NPM1 mut. A and OS and DFS*

**Figure 1:**
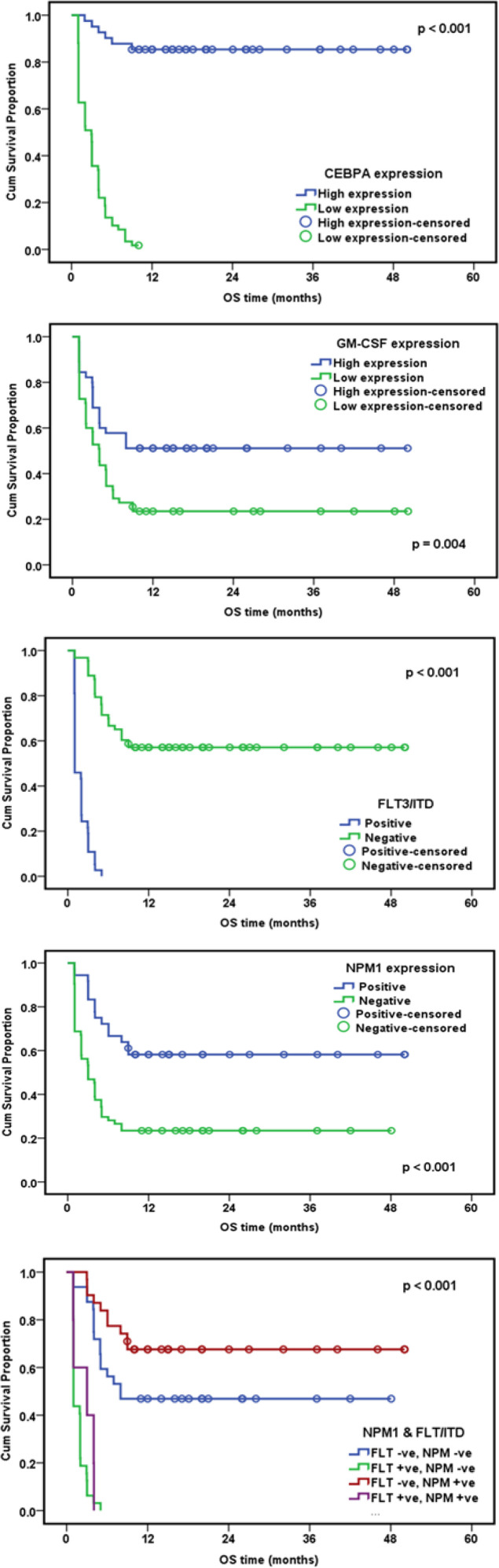
Impact of Studied Molecular Genetic Abnormalities on Overall Survival (OS)

**Figure 2:**
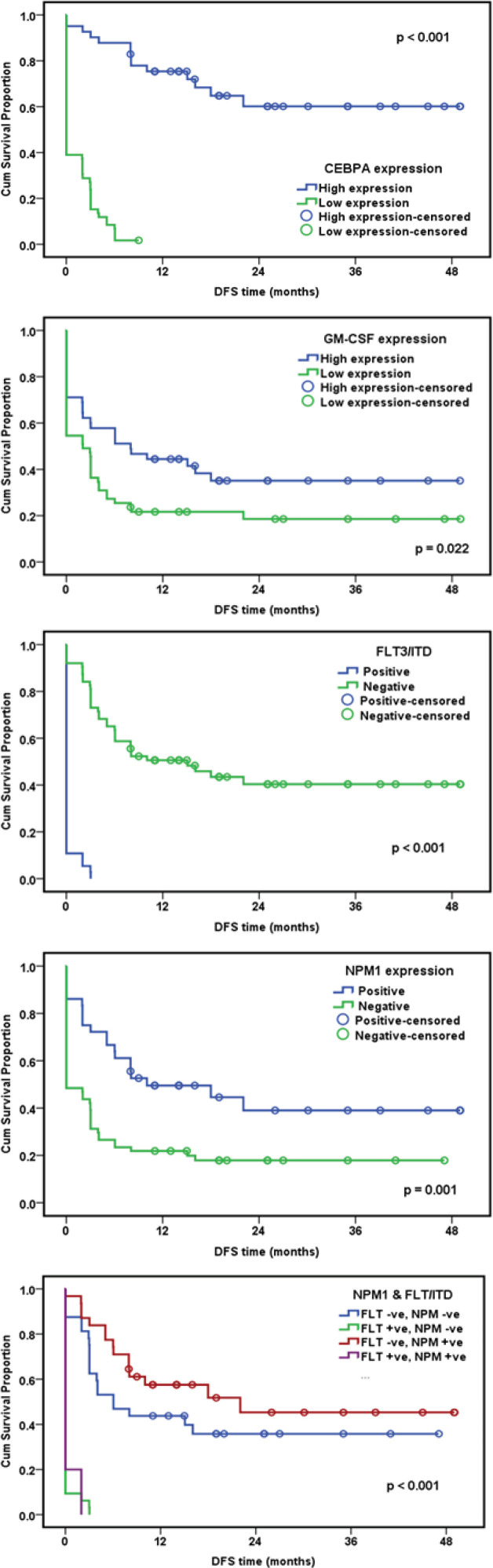
Impact of Studied Molecular Genetic Abnormalities on Disease Free Survival (DFS)

Patients were followed up for a median period of 6 months, the Overall Survival rate (OS: defined from the date of diagnosis till the date the patient died, or was last seen) and the Disease Free Survival rate (DFS: defined from the date of CR achievement till the date the patient relapsed) were assessed. AML patients with high *CEBPA* gene expression had a cumulative OS at 6 months 90.2%, while patients with low *CEBPA* gene expression had a cumulative OS at 6 months 13.6%, with statistically significant difference between the two patients’ group with P value <0.001. Patients with high *GM-CSF* gene expression had a cumulative OS at 6 months 57.8%, while patients with low *GM-CSF* gene expression had a cumulative OS at 6 months 34.5%, with statistically significant difference between the two patients group with P value = 0.004. Regarding *FLT3/ITDs*, a statistically significant difference between wild and mutant cases was noticed with p-value <0.001 where, *FLT3/ITD* wild cases had better OS. As regards *NPM1* mutation A, a statistically significant difference was found between wild and mutant cases with p-value <0.001 where, *NPM1* mutant cases had better OS. Finally, we tried to study the impact of both *FLT3/ITD* and *NPM1* mutations on OS. AML patients were classified into 4 groups where patients with (*FLT3/ITD* –ve, *NPM1* –ve) and (*FLT/ITD3*–ve, *NPM1 *+ve) had higher OS rates than patients with (*FLT3/ITD* +ve, *NPM1* -ve) and (*FLT3/ITD *+ve,* NPM1* +ve) with p-value < 0.001 (Figure 1). Regarding DFS, AML patients with high *CEBPA* gene expression had a cumulative DFS at 6 months 87.8%, while patients with low *CEBPA* gene expression had a cumulative DFS at 6 months of 8.5%, with statistically significant difference encountered between the two patients group with P <0.001. Patients with high *GM-CSF* gene expression had a cumulative DFS at 6 months 57.8%, while patients with low* GM-CSF* gene expression had a cumulative DFS at 6 months of 27.3%, with statistically significant difference between the two patients group with P value = 0.02. Regarding *FLT3/ITDs*, a statistically significant difference between wild and mutant cases was noticed with p-value <0.001 where, *FLT3/ITD* wild cases had better DFS. As regards *NPM1* mutation A, a statistically significant difference was found between wild and mutant cases with p-value = 0.001 where, *NPM1* mutant cases had better DFS. Finally, AML patients were classified into 4 groups where patients with (*FLT3/ITD *–ve, *NPM1* –ve) and (*FLT3/ITD* –ve, *NPM1 *+ve) had higher DFS rates than patients with (*FLT3/ITD* +ve, *NPM1* -ve) and (*FLT3/ITD *+ve,* NPM1* +ve) with p-value < 0.001 (Figure 2).

**Table 1:** Comparison between AML Patients with Low or High* CEBPA* Gene Expression According to Their Clinical and Laboratory Data

Parameter		*CEBPA* low expression			*CEBPA* high expression			*P*-value
		n=59 (59%)			n=41 (41%)			
		Range	Mean±SD	Median	Range	Mean±SD	Median	
Hb gm/dL		5.2-12.4	7.9±1.7	7.7	5.4-15.3	9.0±2.5	8.1	0.03*
TLCx10³/cm³		1.5-272	50,6±50,9	36,6	2.3-419	60,2±67,3	44.5	0.17
Plts x10³/cm³		6-429	78.2±101.3	45	8-337	120.6±103.7	77	0.01*
P.B blast (%)		4-94	42.9±27.1	35	4-90	45.7±28.4	45	0.81
B.M blast (%)		20-90	66.0±22.6	70	22-90	58.2±25.1	60	0.14
Age	> 18 years	57 (60.0%)			38 (40.0%)			0.39
	< 18 years	2 (40.0 %)			3 (60.0 %)			
Gender	Male	27 (56.3%)			21 (43.8%)			0.59
	Female	32 (61.5%)			20 (38.5%)			
FAB	M0,M 1, M2	28 (68.3%)			13 (31.7%)			0.02*
Classification	M3	10 (37.0%)			17 (63.0%)			
	M4,M5&M6,M7	21 (65.6%)			11 (34.4%)			
	Non M3	49 (67.1%)			24 (32.9%)			0.01*
	M3	10 (37.0%)			17 (63.0%)			
Mol. studies	*FLT3-ITD* wild	23 (36.5%)			40 (63.5%)			0.00*
	*FLT3-ITD* mutant	36 (97.3%)			1 (2.7%)			
	*NPM1 mut. A* Wild	44 (68.8%)			20 (31.3%)			0.01*
	*NPM1 mut. A *mutant	15 (41.7%)			21 (58.3%)			
Cytogenetic studies	t (8;21) positive	10(66.7%)			5 (33.3%)			0.51
	t (8;21) negative	49(57.6%)			36 (42.4%)			
	inv.16 positive	16 (64.0%)			9 (36.0%)			0.56
	inv.16 negative	43 (57.3%)			32 (42.7%)			
	t (15;17) positive	11 (40.7%)			16 (59.3%)			0.02*
	t (15;17) negative	48 (65.8%)			25 (34.2%)			

*, Significant at P ≤ 0.05

**Table 2:** Comparison between AML Patients with Low or High *GM-CSF* Gene Expression According to Their Clinical and Laboratory Data

Parameter		*GM-CSF* low expression			*GM-CSF* high expression			P-value
		n=55 (55%)			n=45 (45%)			
		Range	Mean±SD	Median	Range	Mean±SD	Median	
Hb gm/dL		5.2-14.1	8.2±2.2	7.8	5.9-15.3	8.5±2.0	7.9	0.45
TLCx10³/cm³		1.5-419	60,2±71.5	44,7	5.9-164	47,6±35,0	40,0	0.931
Plts x10³/cm³		6-429	89.3±103.5	54	8-402	103.2±105.0	63	0.359
P.B blast (%)		4-90	44.3±29.4	37	5-94	43.8±25.4	44	0.983
B.M blast (%)		20-90	64.0±23.9	66	22-90	61.4±24.0	70	0.602
Age	> 18 years	52 (54.7%)			43 (45.3%)			1
	< 18 years	3 (60.0%)			2 (40.0%)			
Gender	Male	26 (54.2%)			22 (45.8%)			0.87
	Female	29 (55.8%)			23 (44.2%)			
FAB	M0,M 1, M2	22 (53.7%)			19 (46.3%)			0.5
Classification	M3	13 (48.1%)			14 (51.9%)			
	M4,M5&M6,M7	20 (62.5%)			12 (37.5%)			
	Non M3	42 (57.5%)			31 (42.5%)			0.4
	M3	13 (48.1%)			14 (51.9%)			
Mol. studies	*FLT3-ITD* wild	29 (46.0%)			34 (54.0%)			0.02*
	*FLT3-ITD* mutant	26 (70.3%)			11 (29.7%)			
	*NPM1 mut. A* Wild	41 (64.1%)			23 (35.9%)			0.015*
	*NPM1 mut. A* mutant	14 (38.9%)			22 (61.1%)			
Cytogenetic studies	t (8;21) positive	7 (46.7%)			8 (53.3%)			0.5
	t (8;21) negative	48 (56.5%)			37 (43.5%)			
	inv.16 positive	15 (60.0%)			10 (40.0%)			0.6
	inv.16 negative	40 (53.3%)			35 (46.7%)			
	t (15;17) positive	14 (51.9%)			13 (48.1%)			0.7
	t (15;17) negative	41 (56.2%)			32 (43.8%)			

*, Significant at P ≤ 0.05

**Table 3:** Impact of Studied Molecular Genetic Abnormalities on Response to Induction Therapy

	Complete remission (CR)	Resistant disease (RD)	*P*- value
	n=28	n=27	
High *CEBPA* expression	27 (96.4%)	12 (44.4%)	<0.001*
Low *CEBPA* expression	1 (3.6%)	15 (55.6%)	
High* GM-CSF* expression	17 (60.7%)	10 (37%)	0.08
Low *GM-CSF* expression	11 (39.3%)	17 (63%)	
*FLT3/ITD* +ve	0 (0%)	3 (11.1%)	0.11
*FLT3/ITD* –ve	28 (100%)	24 (88.9%)	
*NPM1* +ve	16 (57.1%)	11 (40.7%)	0.22
*NPM1* –ve	12 (42.9%)	16 (59.3%)	
*FLT3/ITD* -ve, *NPM1* +ve	16 (57.1%)	10 (37%)	0.21
*FLT3/ITD* -ve , *NPM1* -ve	12 (42.9%)	14 (51.9%)	
*GM-CSF* high expression, *CEBPA* high expression	17 (100%)	6 (60%)	0.01*
*GM-CSF* high expression, *CEBPA* low expression	0 (0%)	4 (40%)	
*GM-CSF* low expression, *CEBPA* high expression	10 (90.9%)	6 (35.3%)	0.01*
*GM-CSF* low expression, *CEBPA* low expression	1 (9.1%)	11 (64.7%)	

*, Significant at P ≤ 0.05

## Discussion

Acute myeloid leukemia is a group of hematological malignancies whose leukemogenesis and clinical behavior were deeply influenced by the underlying cytogenetic and molecular abnormalities (Zhu et al., 2017). Here, we aim to investigate *GM-CSF *gene expression using quantitative RT-PCR as *GM-CSF* is a known autocrine/paracrine cytokine that stimulates growth, differentiation, andfunction of normal and leukemic myeloid progenitors together with different molecular prognostic markers such as *FLT3/ITD, NPM1* mutation A and *CEBPA* gene expression in Egyptian AML patients. In addition to response to therapy, DFS and OS in these patients were assessed which help in understanding their impact on the pathogenesis of the disease and hence predict prognosis. Our results showed statistically significant difference between low and high *GM-CSF* gene expression levels in AML patients with p value < 0.001. No significant difference was found between low andhigh *GM-CSF* gene expression as regards their age, gender, clinical data, total leukocytic count, and initial peripheral blood blasts percentage. This is in agreement with previously reported by (Kassem et al., 2018) who found no significant correlations between *GM-CSF* gene expression and different demographic, clinical and laboratory data. As regards cytogenetic analysis, we found no statistically significant difference between low and high *GM-CSF* gene expression as regards different cytogenetic markers. This is in disagreement with (Weng et al., 2017) who found *GM-CSF* gene significantly downregulated in t(8;21) positive leukemic patients. Our results revealed no statistically significant difference between low andhigh *GM-CSF* gene expression regarding the response to therapy which in agreement with (Kassem et al., 2018) who found no significant correlations between *GM-CSF *gene expression and response to treatment. Our AML patients with high *GM-CSF* expression levels had better OS and DFS with statistically significant difference between high andlow *GM-CSF* gene expression group, p value 0.004 and 0.02, respectively. This is discordance with (Kassem et al., 2018) who found no significant correlations between* GM-CSF* gene expression and OS. The CCATT enhancer binding protein alpha (*CEBPA*) transcription factor is a critical regulator of proliferation and differentiation in myeloid cells (Zhang et al., 2004). Quantitative assessment of *CEBPA* gene expression was done by real time PCR and our study showed that there was a statistically significant difference between low and high *CEBPA* gene expression where the majority of patients had low *CEBPA *expression levels. This is in accordance with the results previously reported by (Barjesteh et al., 2003; D’Al`o et al., 2008) who reported that the majority of their patients showed low *CEBPA* expression level. Also, we found no statistically significant difference between low andhigh *CEBPA* gene expression as regards their gender. However, Gholami et al., (2019) found that a significant up-regulation of *CEBPA* gene was detected in male AML patients. Also, we found no statistically significant difference between low andhigh* CEBPA* gene expression as regards their clinical andlaboratory data except for hemoglobin (Hb) and platelet count where patients with high *CEBPA* expression levels had significant higher Hb andplatelet counts. However, Gholami et al., (2019) found patients with a lower level of *CEBPA* gene expression had leukopenia. Our results revealed that M3 patients had significant higher *CEBPA* gene expression levels than non M3 patients with p-value 0.01. This is in accordance with (Kassem et al., 2013; Gholami et al., 2019) who found significant *CEBPA* gene over-expression was in M3. Regarding cytogenetic analysis, our results showed no statistically significant difference between low andhigh* CEBPA* gene expression but most of low *CEBPA* expression patients harboring t(8;21). This is in accordance to (Grossmann et al., 2012) who found cases harboring t(8;21) presented a lower *CEBPA* expression than patients without, where no significant difference was detected between *CEBPA* expression levels and *RUNX1 *mutations. Our results showed statistically significant difference between patients with high andlow *CEBPA *gene expression levels as regards the response to therapy with most of patients with low *CEBPA *expression having resistant disease together with poor OS and DFS. This is in accordance with (Kassem et al., 2013) who found patients with higher *CEBPA* gene expression had higher OS and DFS and (Barjesteh et al., 2003) who found that particularly patients with low *CEBPA* expression seemed to have a relatively poor OS and DFS but didn’t reach significant difference. Our results showed that AML patients with high *CEBPA* gene expression whether *GM-CSF* gene expression was high or low had significant higher complete remission rates. However, low* GM-CSF* gene expression andlow* CEBPA *gene expression showed poor response to treatment. We also assessed *NPM1* mutation A and *FLT3/ITDs* by conventional RT-PCR because of their known prognostic value besides being potential targets for therapy. *NPM1* mutation A was detected in 36% of AML patients. This frequency was in agreement with many previous studies in Egypt and worldwide as reported by (Falini et al., 2008; Farawela et al., 2014; Kassem et al., 2019) where the frequency of *NPM1* mutation ranged between 30–52.9% among their AML patients. *FLT3/ITDs* was detected in 37% of our AML patients, this frequency was close to that previously reported by (Gorin et al., 2013; Farawela et al., 2014; Kassem et al., 2019) where the frequency of *FLT3/ITDs* ranged between 15.4–36% among their AML patients. Our results revealed no statistically significant difference between *NPM1* mut. A and *FLT3/ITD* wild and mutant patients regarding response to therapy. This is in accordance to (Akla et al., 2012) where no significant difference was detected between *FLT3/ITD *positive andnegative cases after induction chemotherapy. Although, he recorded a significant difference between *NPM1*-positive and -negative patients with a P- value 0.004 where 62.5% of *NPM1*-positive patients achieved CR. We classified our patients regarding *NPM1* mut. A and *FLT3/IT*D mutational status into 2 subgroups (*NPM1* +ve, *FLT/ITD* -ve and *NPM1* –ve, *FLT3/ITD* -ve). Fifty seven percent of our patients who achieved CR were in (*FLT3/ITD *–ve, *NPM1* +ve) group. This in accordance to (Akla et al., 2012; Testa and Pelosi 2013) who found that 60% who achieved CR were *NPM1+/FLT3/ITD* – and had favorable outcome. Regarding DFS and OS, there was a statistically significant difference between *FLT3/ITD* mutant and wild patients with p-value < 0.001 for each, where *FLT3/ITD *wild cases had better DSF and OS. This is in agreement with (Medinger et al., 2016; Garcia and Baer, 2017) as they reported that *FLT3ITD*-positive AML patients had higher relapse incidence and lower DFS as well as OS. A statistically significant difference was detected between *NPM1* mutant and wild cases as regards DFS and OS with p-value 0.001 and< 0.001, respectively. Most of patients with positive *NPM1* mutation A had better DFS and OS. This is in accordance with (Port et al., 2014) who found a better outcome for DFS and OS for patients harboring *NPM1* mutations. In the current study, we classified our patients into 4 groups as regards *FLT3-ITD* and *NPM1 *mutations where higher DFS and OS were detected in (*NPM1* mutant,* FLT3/ITD* wild) group .This is in accordance to (Medinger et al., 2016; Velloso et al., 2011) who found that absence of *FLT3 ITD* mutations, positive *NPM1* mutations are associated with improved outcome for patients and *NPM1+/FLT3*-, currently recognized as of good prognosis.

In conclusion, this study identified *GM-CSF* gene expression in AML patients providing additional evidence for the possible role of that gene as a prognostic marker and indicator for treatment outcome together with already known prognostic molecular biomarkers such as *CEBPA, NPM1* mut. A and *FLT3-ITD*. Additional researches in this field with larger sample size involving the majority of oncology centers throughout Egypt’s governorates are required for understanding the molecular mechanisms underlying AML pathogenesis and risk stratification in Egypt. In conclusion, our findings suggest that molecular diagnostic biomarkers for AML are an essential tool that improves prognostication andhence better patients’ management. 
